# CSF Rhinorrhoea After Endonasal Intervention to the Skull Base (CRANIAL) - Part 1: Multicenter Pilot Study

**DOI:** 10.1016/j.wneu.2020.12.171

**Published:** 2021-05

**Authors:** CRANIAL Consortium, Danyal Z. Khan, Hani J. Marcus, Hugo Layard Horsfall, Soham Bandyopadhyay, Benjamin E. Schroeder, Vikesh Patel, Alice O’Donnell, Shahzada Ahmed, Andrew F. Alalade, Ahmad M.S. Ali, Callum Allison, Sinan Al-Barazi, Rafid Al-Mahfoudh, Meriem Amarouche, Anuj Bahl, David Bennett, Raj Bhalla, Pragnesh Bhatt, Alexandros Boukas, Ivan Cabrilo, Annabel Chadwick, Yasir A. Chowdhury, David Choi, Simon A. Cudlip, Neil Donnelly, Neil L. Dorward, Graham Dow, Daniel M. Fountain, Joan Grieve, Anastasios Giamouriadis, Catherine Gilkes, Kanna Gnanalingham, Jane Halliday, Brendan Hanna, Caroline Hayhurst, Jonathan Hempenstall, Duncan Henderson, Kismet Hossain-Ibrahim, Theodore Hirst, Mark Hughes, Mohsen Javadpour, Alistair Jenkins, Mahmoud Kamel, Richard J. Mannion, Angelos G. Kolias, Mohammad Habibullah Khan, Mohammad Saud Khan, Peter Lacy, Shumail Mahmood, Eleni Maratos, Andrew Martin, Nijaguna Mathad, Patrick McAleavey, Nigel Mendoza, Christopher P. Millward, Showkat Mirza, Sam Muquit, Daniel Murray, Paresh P. Naik, Ramesh Nair, Claire Nicholson, Alex Paluzzi, Omar Pathmanaban, Dimitris Paraskevopoulos, Jonathan Pollock, Nick Phillips, Rory J. Piper, Bhaskar Ram, Iain Robertson, Elena Roman, Peter Ross, Thomas Santarius, Parag Sayal, Jonathan Shapey, Rishi Sharma, Simon Shaw, Alireza Shoakazemi, Syed Shumon, Saurabh Sinha, Georgios Solomou, Wai Cheong Soon, Simon Stapleton, Patrick Statham, Benjamin Stew, Nick Thomas, Georgios Tsermoulas, James R. Tysome, Adithya Varma, Philip Weir, Adam Williams, Mohamed Youssef

**Affiliations:** 1 Wellcome / EPSRC Centre for Interventional and Surgical Sciences & National Hospital for Neurology and Neurosurgery, London, United Kingdom; 2 Medical Sciences Division, University of Oxford, Oxford, United Kingdom; 3 School of Medicine, Cardiff University, Cardiff, United Kingdom; 4 Division of Neurosurgery, Department of Clinical Neurosciences, Cambridge University Hospitals Trust, Cambridge, United Kingdom; 5 Birmingham Medical School, University of Birmingham, Birmingham, United Kingdom; 6 Department of Ear, Nose & Throat (ENT), University Hospitals Birmingham NHS Foundation Trust, Birmingham, United Kingdom; 7 Department of Neurosurgery, Lancashire Teaching Hospitals NHS Foundation Trust, Chorley, United Kingdom; 8 Department of Neurosurgery, The Walton Centre, Liverpool, United Kingdom; 9 Department of Neurosurgery, Royal Victoria Infirmary, Newcastle, United Kingdom; 10 Department of Neurosurgery, King's College Hospital, London, United Kingdom; 11 Department of Neurosurgery, Hurstwood Park Neurosciences Centre and Royal Sussex County Hospital, Haywards Heath, United Kingdom; 12 Department of Neurosurgery, John Radcliffe Hospital, Oxford University Hospitals NHS Foundation Trust, Oxford, United Kingdom; 13 Department of Neurosurgery, Hull University Teaching Hospitals, Hull, United Kingdom; 14 Department of Neurosurgery, Ninewells Hospital, Dundee, United Kingdom; 15 Department of ENT, Salford Royal NHS Foundation Trust, Salford, United Kingdom; 16 Department of Neurosurgery, Aberdeen Royal Infirmary, Aberdeen, United Kingdom; 17 National Hospital for Neurology and Neurosurgery, London, United Kingdom; 18 Department of Neurosurgery, Manchester Centre for Clinical Neurosciences, Salford Royal Trust, Manchester, United Kingdom; 19 Department of Neurosurgery, Queen Elizabeth Hospital Birmingham, Birmingham, United Kingdom; 20 ENT Department, Cambridge University Hospitals Trust, Cambridge, United Kingdom; 21 Department of Neurosurgery, Queen's Medical Centre Nottingham, Nottingham, United Kingdom; 22 Department of ENT, Royal Victoria Hospital, Belfast, United Kingdom; 23 Department of Neurosurgery, University Hospital of Wales, Cardiff, United Kingdom; 24 Department of Neurosurgery, University Hospital Southampton, Southampton, United Kingdom; 25 Department of Neurosurgery, Royal Hallamshire Hospital & Sheffield Children’s Hospital, Sheffield, United Kingdom; 26 Department of Neurosurgery, Royal Victoria Hospital, Belfast, United Kingdom; 27 Department of Neurosurgery, The Western General Hospital, Edinburgh, United Kingdom; 28 Department of Neurosurgery, National Neurosurgical Centre, Beaumont Hospital, Dublin, Ireland; ^29^Department of Neurosurgery, Cork University Hospitals, Cork, Ireland; 30 Department of ENT, Cork University Hospitals, Cork, Ireland; 31 Department of ENT, National Neurosurgical Centre, Beaumont Hospital, Dublin, Ireland; 32 Department of Neurosurgery, St George’s University Hospitals Trust, London, United Kingdom; 33 Department of Neurosurgery, University Hospital Southampton, United Kingdom; 34 Department of Neurosurgery, Charing Cross Hospital, London, United Kingdom; 35 Department of ENT, Sheffield Teaching Hospitals, Sheffield, United Kingdom; 36 Department of Neurosurgery, University Hospitals Plymouth, Plymouth, United Kingdom; 37 Department of Neurosurgery, Barts and The Royal London Hospital, London, United Kingdom; 38 Department of Neurosurgery, Barking, Havering & Redbridge University Hospitals, Romford, United Kingdom; 39 Department of ENT, Aberdeen Royal Infirmary, Aberdeen, United Kingdom; 40 Department of ENT, Ninewells Hospital, Dundee, United Kingdom; 41 Department of Neurosurgery, Royal Stoke University Hospital, Stoke-on-Trent, United Kingdom; 42 School of Medicine, Keele University, Stoke-on-Trent, United Kingdom; 43 Department of ENT, University Hospital of Wales, Cardiff, United Kingdom; 44 ENT Department, Cambridge University Hospitals Trust, Cambridge, United Kingdom; 45 Department of Neurosurgery, Southmead Hospital Bristol, Bristol, United Kingdom; 46 Department of Neurosurgery, Leeds Teaching Hospitals NHS Trust, Leeds, United Kingdom

**Keywords:** Cerebrospinal fluid leak, Cerebrospinal fluid rhinorrhea, CSF, EEA, Endoscopic endonasal, Skull base surgery, BMI, Body mass index, CRANIAL, CSF Rhinorrhoea After Endonasal Intervention to the Skull Base, CSF, Cerebrospinal fluid, EEA, Expanded endonasal approach, TSA, Transsphenoidal approach

## Abstract

**Background:**

CRANIAL (CSF Rhinorrhoea After Endonasal Intervention to the Skull Base) is a prospective multicenter observational study seeking to determine 1) the scope of skull base repair methods used and 2) corresponding rates of postoperative cerebrospinal fluid (CSF) rhinorrhea in the endonasal transsphenoidal approach (TSA) and the expanded endonasal approach (EEA) for skull base tumors. We sought to pilot the project, assessing the feasibility and acceptability by gathering preliminary data.

**Methods:**

A prospective observational cohort study was piloted at 12 tertiary neurosurgical units in the United Kingdom. Feedback regarding project positives and challenges were qualitatively analyzed.

**Results:**

A total of 187 cases were included: 159 TSA (85%) and 28 EEA (15%). The most common diseases included pituitary adenomas (*n* = 142/187), craniopharyngiomas (*n* = 13/187). and skull base meningiomas (*n* = 4/187). The most common skull base repair techniques used were tissue glues (*n* = 132/187, most commonly Tisseel), grafts (*n* = 94/187, most commonly fat autograft or Spongostan) and vascularized flaps (*n* = 51/187, most commonly nasoseptal). These repairs were most frequently supported by nasal packs (*n* = 125/187) and lumbar drains (*n* = 20/187). Biochemically confirmed CSF rhinorrhea occurred in 6/159 patients undergoing TSA (3.8%) and 2/28 patients undergoing EEA (7.1%). Four patients undergoing TSA (2.5%) and 2 patients undergoing EEA (7.1%) required operative management for CSF rhinorrhea (CSF diversion or direct repair). Qualitative feedback was largely positive (themes included user-friendly and efficient data collection and strong support from senior team members), demonstrating acceptability.

**Conclusions:**

Our pilot experience highlights the acceptability and feasibility of CRANIAL. There is a precedent for multicenter dissemination of this project, to establish a benchmark of contemporary practice in skull base neurosurgery, particularly with respect to patients undergoing EEA.

## Introduction

The endonasal transsphenoidal approach (TSA) has developed into the approach of choice for resecting pituitary adenoma and most sellar masses.^1^^,^^2^ More recently, the expanded endonasal approach (EEA) has bolstered endoscopic access to the skull base, allowing resection of many diseases extending beyond the sella alone, including large pituitary adenomas, craniopharyngiomas, Rathke cleft cysts, meningiomas, and clival chordomas.^3^^,^^4^ Despite the benefits that these minimally invasive approaches afford, cerebrospinal fluid (CSF) rhinorrhea remains a frequent complication,^5^, ^6^, ^7^ with potentially serious consequences, including meningitis, pneumocephalus, low-pressure headaches, and prolonged admission.^6^^,^^8^^,^^9^

Arguably, the most important determinant for the development of CSF rhinorrhea is the skull base repair technique used intraoperatively.^4^ Other risk factors for postoperative CSF rhinorrhea include previous cranial radiotherapy or surgery; tumor size and infiltration; high-flow intraoperative CSF leak; dural defect size; increased body mass index (BMI, calculated as weight in kilograms divided by the square of height in meters); and surgeon experience.^4^^,^^5^^,^^7^^,^^10^, ^11^, ^12^ A vast array of options and combinations is available for repairing the skull base, including direct closure of the dura using sutures or clips; dural reconstruction using autologous fascia or synthetic materials; vascularized flaps (e.g. nasoseptal and turbinate flaps); avascular grafts (e.g. fat grafts); synthetic grafts; and tissue glues (e.g. fibrin glues).^4^^,^^12^, ^13^, ^14^, ^15^ These repair constructs are often supported by buttresses (e.g. septal bone or titanium mesh), nasal packing (e.g. Merocel packs [Medtronic Inc., Minneapolis, Minnesota, USA]), and lumbar drains.^4^^,^^13^^,^^14^ The choice of repair can be graded in response to numerous factors, such as tumor (type, size, hydrocephalus), defect (size, extent of intraoperative arachnoid breach), patient (BMI, sinonasal disease) and operation (approach, primary or revision).^14^^,^^16^ Previous observational studies suggest that there may be a role for nasoseptal flaps in the context of high-grade intraoperative CSF leak (high-flow leaks with large dural defects).^17^^,^^18^ In addition, a recent randomized controlled trial suggests that perioperative lumbar drain use combined with nasoseptal flap repair (in the context of dural defects >1 cm^2^ and high-flow intraoperative CSF leak), significantly decreases CSF rhinorrhea rates.^19^ However, overall, there is a lack of comparative evidence and consensus as to the optimal reconstruction technique; this is the case in high-flow and low-flow intraoperative CSF leaks, small and large dural defects, and primary and revision surgery.^14^ Resultantly, there is considerable heterogeneity in skull base repair protocols (largely based on surgeon opinion)^14^ with complementary variations in CSF rhinorrhea rates: generally up to 5% for TSA and generally up to 20% for EEA (although as high as 50% in some EEA case series).^4^^,^^7^^,^^8^^,^^20^, ^21^, ^22^, ^23^

CRANIAL (CSF Rhinorrhoea After Endonasal Intervention to the Skull Base) is a prospective multicenter observational study seeking to determine 1) the scope of the methods of skull base repair and 2) the corresponding rates of postoperative CSF rhinorrhea in contemporary neurosurgical practice in the United Kingdom and Ireland.^24^ The project is a collaboration between 3 principal bodies: students and junior doctors via NANSIG (Neurology and Neurosurgery Interest Group), neurosurgical specialty trainees via the BNTRC (British Neurosurgical Trainee Research Collaborative), and skull base consultants (neurosurgery and ear nose and throat) via the CRANIAL steering committee. Thus far, 29 centers (of the 40 adult and pediatric neurosurgical centers in the United Kingdom and Ireland) have been recruited to join the project, with each center having a local team of consultants, trainees, junior doctors, and medical students.

Before national dissemination, the project was piloted at selected centers. The usefulness of piloting multicenter studies before scaling is well established and includes assessing protocol feasibility, logistic planning, refining data collection and recruitment instruments, and increasing the investment of key stakeholders.^25^ The CONSORT (Consolidated Standards of Reporting Trials) statement^26^ has recently extended its guidelines to include feasibility projects, recognizing their role in refining methodologies and processes before definitive multicenter studies. In the context of previous BNTRC studies, reflection on pilot experiences has proved formative in streamlining recruitment, study setup, and data collection before expansion.^27^

In this article, the feasibility, acceptability, and practicality of the proposed CRANIAL study are assessed. We present preliminary data collected and outline our experience, the successes, and the challenges in establishing a scalable version of the CRANIAL study.

## Methods

### Design

**Figure 1:**
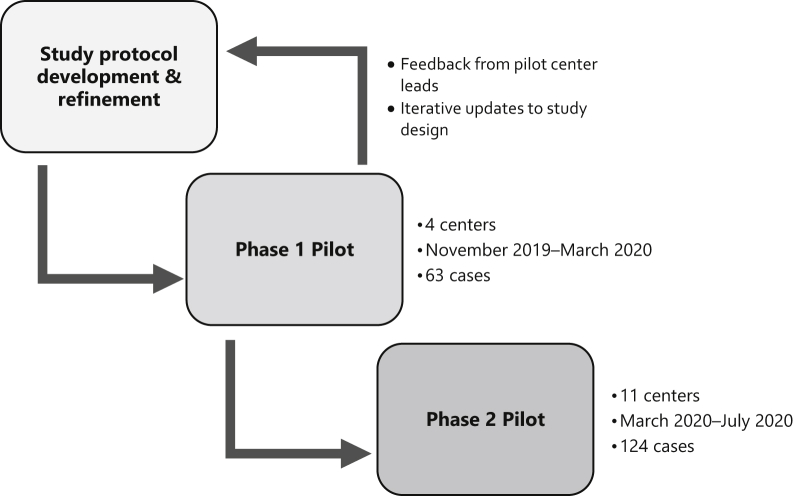
Study case flowchart.

A multicenter prospective observational cohort study design was implemented across multiple tertiary academic neurosurgical units in 2 phases.^28^ Phase 1 (November 1, 2019–March 22, 2020) represented nonconsecutive case recruitment at Addenbrooke's Hospital (Cambridge, United Kingdom), John Radcliffe Hospital (Oxford, United Kingdom), National Hospital for Neurology and Neurosurgery (London, United Kingdom), and Queen Elizabeth Hospital (Birmingham, United Kingdom) (Figure 1). Phase 2 (March 23, 2020–July 31, 2020) represented upscaling of the study across 12 centers for consecutive case selection: Aberdeen Royal Infirmary (Aberdeen, United Kingdom), Addenbrooke's Hospital (Cambridge, United Kingdom), Beaumont Hospital (Dublin, Ireland), Greater Manchester Neurosciences Centre (Salford, United Kingdom), John Radcliffe Hospital (Oxford, United Kingdom), National Hospital for Neurology and Neurosurgery (London, United Kingdom), Royal Hallamshire Hospital (Sheffield, United Kingdom), Royal Victoria Hospital (Belfast, United Kingdom), Royal Victoria Infirmary (Newcastle-upon-Tyne, United Kingdom), Sheffield Children's Hospital (Sheffield, United Kingdom), and the Walton Centre (Liverpool, United Kingdom). The project was registered as a service evaluation at each center, garnering approvals from audit departments (and Caldicott guardians when required). The local team consisted of consultant lead(s) with overall project responsibility, trainee lead(s) in charge of data collection, and on occasion, student lead(s) for additional support. The STROBE (Strengthening the Reporting of Observational Studies in Epidemiology) statement was used in the preparation of this article.^29^

Eligible patients included those of all ages undergoing TSA for sellar tumors and EEA for skull base tumors.^28^ The TSA was defined as surgical access to the sella alone (transsphenoidal), whereas the EEA was defined as acquiring surgical access to an area beyond the sella (e.g. transtubercular or transclival).^24^ Exclusion criteria were patients undergoing transcranial surgery and those with a history of preoperative CSF rhinorrhea. Case selection was nonconsecutive because of pauses in collection for data proforma amendments and attaining extra approvals (e.g. information governance approvals when requested).

### Data Collection

Data points collected were patient demographics, tumor characteristics, operative data, and postoperative outcomes.^24^ Dural defects were recorded as <1 cm, 1–3 cm, or >3 cm,^17^ and intraoperative CSF leak grade was recorded as grade 0 (small leak without obvious diaphragmatic defect), grade 1 (small leak with a small diaphragmatic defect), grade 2 (moderate leak with obvious diaphragmatic defect), or grade 3 (large leak typically created as a part of EEA).^16^ Primary outcomes were 1) methods of intraoperative skull base reconstruction used and 2) postoperative CSF rhinorrhea requiring intervention (CSF diversion and/or operative repair).

Local teams submitted data to a secure Web-based central database hosted by Castor Electronic Data Capture (https://www.castoredc.com/). All data were collected within 30 days of operation. Data points collected by medical students or junior trainees were confirmed with operating surgeons or senior members of the team before final submission into the Castor Electronic Data Capture system.^24^ To facilitate accurate and standardized discussion of skull base repair techniques, supportive materials were provided: skull base repair taxonomy, illustrations, and clear definitions.^24^

In addition to the data outlined earlier, qualitative data were collected from local pilot trainee leads with an open question “Tell us about your experience during the CRANIAL project – the positives and challenges.” This information, along with the procedural experience of the management committee overseeing the project, informed a set of iterative changes to the project.

### Data Validation

Data validation was performed in all 3 centers to audit quantitative data accuracy. This process involved an independent data validator (who did not collect local data) who reviewed data sets for several enrolled cases, selected randomly. This data validator was from the hospital in which the data were collected. The targets for validation were a secure and accurate record of Castor identification records with corresponding medical record numbers; no case/data duplication; and data accuracy >95%.

### Data Analysis

Pooled quantitative data (from phase 1 and 2) were analyzed using Microsoft Excel version 16.41 (Microsoft, Redmond, Washington, USA) to present descriptive statistics. The data were used to create tables summarizing demographic, tumor, and operative characteristics. Tumor characteristics, intraoperative skull base repair technique, and dural defect size with associated intraoperative CSF leak grade are graphically shown. Six months follow-up data were not available or complete in most cases because of the recency of data collection and were excluded. Qualitative feedback from local pilot leads from the phase 1 centers was analyzed in terms of content using NVivo software (version 12.6.0). Deductive coding was performed by an independent author (D.Z.K.). Codes were used to generate themes, which in turn were organized into the following categories according to content analysis: 1) pilot positives and 2) pilot challenges.

## Results

Data were collected on 187 patients across the tertiary neurosurgical centers, between November 2019 and July 2020 inclusive (Figure 1). There were no duplicates in cases/data in the records audited for data validation. All centers fulfilled the >95% accuracy target per case.

### Patient Characteristics

The median age of patients within the study was 52 years (range, 7–84). There were 95 male patients and 92 female patients. At presentation, BMI was recorded in 182 patients (*n* = 182/187, 97%). Fifty-five patients (*n* = 55/182, 30%) had a BMI >30 (TSA: 50/159, 31%; EEA: 5/28, 18%). The patient's vision at presentation was recorded in 185 patients (*n* = 185/187, 99%). Visual loss (acuity and/or field deficits) was present in 107 patients (*n* = 107/185, 58%) preoperatively (TSA: 89/159 56%; EEA: 18/28, 64%). Forty-four patients (*n* = 44/187, 24%) presented with anterior pituitary deficiency requiring hydrocortisone preoperatively (TSA: 38/159, 24%; EEA: 6/28, 21%). Six patients (*n* = 6/187, 3%) had posterior pituitary deficiency requiring desmopressin preoperatively (TSA: 5/159, 3%; EEA: 1/28, 4%). This information is summarized in Table 1.

**Table 1:** Summary of Patient Demographics on Admission and Operation Characteristics per Approach Subgroup

Approach	Transsphenoidal Approach	Expanded Endonasal Approach	Total
Total number of patients	159	28	187
Preoperative data			
Age (years), median (range)	51 (10–84)	55 (7–76)	52 (7–84)
Males	81	14	95
Females	78	14	92
Body mass index			
>30 kg/m^2^	50	5	55
<30 kg/m^2^	104	23	127
Preoperative visual loss	89	18	107
No preoperative visual loss	69	9	78
Anterior pituitary deficiency requiring hydrocortisone	38	6	44
Posterior pituitary deficiency requiring desmopressin	5	1	6
Operative data			
Specialty performing: neurosurgery only	107	10	117
Specialty performing: neurosurgery and ENT	47	17	64
Specialty performing: ENT only	5	1	6
Endoscopic technique	134	28	162
Microscopic endoscopic	25	—	25
Neuronavigation use	61	22	83
Operation time (minutes), median (range)	89 (30–512)∗	192 (64–433)	99 (30–512)

ENT, ear nose, and throat.

∗ The outlying transsphenoidal approach case, which took 512 minutes, was an invasive sinonasal cancer that had infiltrated the sella and required concomitant extracranial resection.

Most tumors were pituitary adenomas (*n* = 142/187, 76%), mostly macroadenomas (*n* = 132/142, 93%). There were 96 nonfunctioning pituitary adenomas (*n* = 96/142, 68%), of which 95 were macroadenomas (*n* = 95/96, 99%). Of the functioning pituitary adenomas (*n* = 46/142, 32%), 30 were macroadenomas (*n* = 30/46, 65%). The characteristics of the remaining tumors can be found in Table 2.

**Table 2:** Number of Cases with Each Type of Tumor

Type of Tumor	Transsphenoidal Approach (n)	Expanded Endonasal Approach (n)	Total (n)
Nonfunctioning pituitary adenoma	92	4	96
Functioning pituitary adenoma	45	1	46
Craniopharyngiomas	4	9	13
Meningiomas	0	4	4
Rathke cleft cysts	7	0	7
Apoplexy	2	1	3
Chordomas	1	2	3
Arachnoid cysts	1	1	2
Dermoid cyst	0	1	1
Germinomas	1	0	1
Hypophysitis	1	0	1
Meningoencephalocele	0	1	1
Undefined neuroendocrine tumor	1	0	1
Melanoma metastasis	1	1	2
Prostate metastasis	1	0	1
Lung metastasis	0	1	1
Sinonasal carcinoma	0	1	1
Sinonasal endocrine tumor	1	0	1
Squamous cell carcinoma	0	1	1
Mucinous glands	1	0	1

### Operation Characteristics

Operation characteristics are shown in Table 1. Most cases used TSA (*n* = 159/187, 85%). Of the cases that used TSA, 134 were performed endoscopically (*n* = 134/159, 84%) and 25 were performed microscopically (*n* = 25/159, 16%). The most common tumors operated on via TSA were nonfunctioning pituitary adenoma (92/159, 58%), functioning pituitary adenoma (45/159, 28%), and Rathke cleft cysts (7/159, 4%) (Table 2). EEA was used 28 times (*n* = 28/187, 15%), with the most common tumors operated on being craniopharyngiomas (*n* = 9/28, 32%), meningiomas (*n* = 4/28, 14%), and nonfunctioning pituitary adenomas (*n* = 4/28, 14%) (Table 2).

### Intraoperative CSF Leak and Dural Defects

There were 66 cases (*n* = 66/187, 35%) of intraoperative CSF leak. In 7 cases of intraoperative CSF leak (*n* = 7/66, 11%), arachnoid breach was a planned and necessary part of the operation. Regarding TSA cases, CSF leak was present in 46 cases (*n* = 46/159, 29%) with the following severity grades: grade 1 CSF leak in 23 (*n* = 23/159, 14%), grade 2 leak in 16 cases (*n* = 16/159, 10%), and grade 3 CSF leak in 1 case (*n* = 3/159, 2%). In some cases (*n* = 6/159, 4%), a CSF leak was detected by the operating surgeon, but the grade was unspecified. Regarding EEA cases, most had an intraoperative CSF leak (*n* = 20/28, 71%): grade 1 CSF leak in 1 (*n* = 1/28, 4%), grade 2 leak in 3 cases (*n* = 3/28, 11%), grade 3 CSF leak in 8 cases (*n* = 8/28, 29%), and unspecified in 8 cases (*n* = 8/28, 29%). Most cases of intraoperative CSF leak (*n* = 48/66, 73%) were detected without any intraoperative adjuncts. For 6 cases (*n* = 6/66, 9%), the Valsalva maneuver was performed to detect the CSF leak: all of these being TSA with low-flow (grade 1) leaks. Intrathecal fluorescein was used to detect a CSF leak (unspecified grade) in 1 case (*n* = 1/66, 2%) in which TSA was used.

Intraoperative dural defect maximum diameter was recorded in 111 (*n* = 113/159, 71%) of TSA cases. Among TSA cases, the maximum diameter of the intraoperative dural defect was recorded as <1 cm in 41 cases (*n* = 41/111, 37%), 1–3 cm in 70 cases (*n* = 70/111, 63%), and >3 cm in no cases. Intraoperative dural defect maximum diameter was recorded in 19 (*n* = 19/28, 68%) of EEA cases. Among EEA cases, the maximum diameter of the intraoperative dural defect was recorded as < 1 cm in 4 cases (*n* = 4/19, 21%), 1–3 cm in 11 cases (*n* = 11/19, 58%), and >3 cm in 4 cases (*n* = 4/19, 21%).

### Skull Base Reconstruction and Support

**Figure 2:**
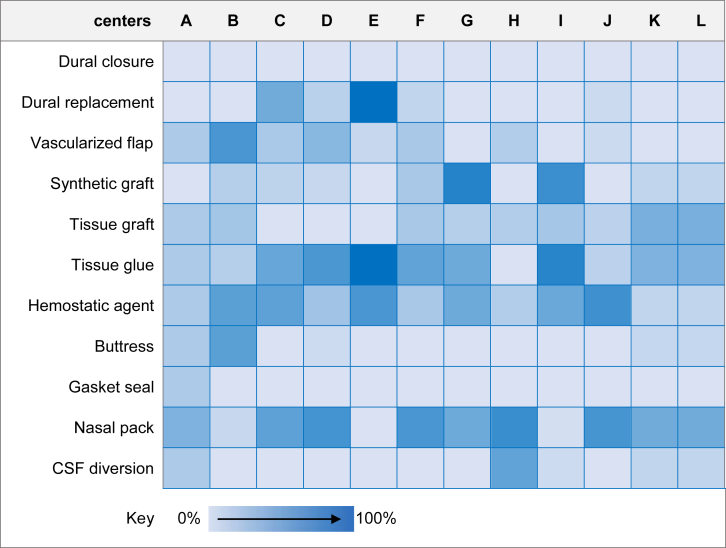
Correlogram showing frequency of repair technique category use per center for transsphenoidal cases. CSF, cerebrospinal fluid.

**Figure 3:**
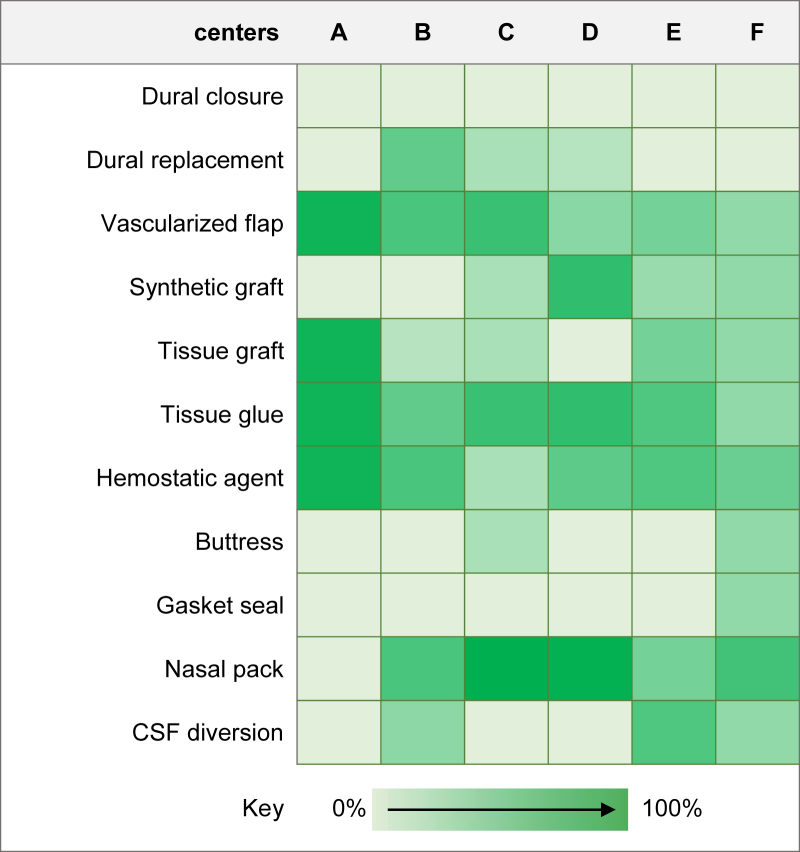
Correlogram highlight frequency of repair technique category use per center for expanded endonasal cases. CSF, cerebrospinal fluid.

Skull base reconstruction included the use of dural repair, dural replacement, glues, hemostatic agents, grafts, and pedicled flaps. Compiled EEA and TSA repair technique frequencies per preoperative and operative risk factors for CSF leak are shown in Table 3. Figures 2 and 3 show the heterogeneity of repair technique frequency per center.

**Table 3:** Repair Technique Categories by Selected Preoperative and Operative Factors

Category	Dural Closure	Dural Replacement	Tissue Graft	Synthetic Graft	Button Technique	Tissue Glue	Hemostatic Agent	Gasket Sealing	Buttress	Pedicled Flap	Nasal Packing	Cerebrospinal Fluid Diversion
	n (% of Category Total)	n (% of Category Total)	n (% of Category Total)	n (% of Category Total)	n (% of Category Total)	n (% of Category Total)	n (% of Category Total)	n (% of Category Total)	n (% of Category Total)	n (% of Category Total)	n (% of Category Total)	n (% of Category Total)
Body mass index (if specified)												
<30 kg/m^2^ (n = 127)	0 (0)	25 (19.8)	42 (33.3)	38 (30.2)	5 (4)	85 (67.5)	75 (59.5%)	3 (2.4)	14 (11.1)	39 (31)	83 (65.9)	4 (3.2)
>30 kg/m^2^ (n = 55)	0 (0)	12 (21.8)	15 (27.3)	17 (30.9)	2 (3.6)	47 (85.5)	26 (47.3%)	0 (0)	5 (9.1)	11 (20)	37 (67.3)	3 (5.5)
Tumor diameter (if specified)												
<1 cm (n = 18)	0 (0)	1 (5.6)	4 (22.2)	5 (27.8)	0 (0)	13 (72.2)	9 (50%)	0 (0)	2 (11.1)	2 (11.1)	10 (55.6)	0 (0)
>1 cm (n = 169)	0 (0)	37 (21.9)	54 (32)	50 (29.6)	7 (4.1)	119 (70.4)	94 (55.6%)	3 (1.8)	17 (10.1)	49 (29)	115 (68)	7 (4.1)
Approach												
Transsphenoidal approach (n = 159)	0 (0)	28 (17.6)	47 (29.6)	45 (28.3)	7 (4.4)	110 (69.2)	82 (51.6%)	1 (0.6)	15 (9.4)	30 (18.9)	99 (62.3)	12 (7.5)
Expanded endonasal approach (n = 28)	0 (0)	10 (35.7)	11 (39.3)	10 (35.7)	0 (0)	22 (78.6)	21 (75%)	2 (7.1)	4 (14.3)	21 (75)	26 (92.9)	10 (35.7)
Intraoperative cerebrospinal fluid leak grade (if specified)												
0 (n = 121)	0 (0)	18 (14.9)	24 (19.8)	33 (27.3)	2 (1.7)	74 (61.2)	67 (55.4%)	1 (0.8)	12 (9.9)	17 (14)	74 (61.2)	2 (1.7)
1 (n = 24)	0 (0)	5 (20.8)	13 (54.2)	8 (33.3)	1 (4.2)	22 (91.7)	9 (37.5)	0 (0)	1 (4.2)	6 (25)	14 (58.3)	1 (4.2)
2 (n = 19)	0 (0)	3 (15.8)	10 (52.6)	5 (26.3)	3 (15.8)	18 (94.7)	8/19 (50%)	0 (0)	4 (21.1)	10 (52.6)	17 (89.5)	1 (5.3)
3 (n = 9)	0 (0)	4 (44.4)	6 (66.7)	3 (33.3)	0 (0)	9 (100)	8 (88.9)	0 (0)	0 (0)	8 (88.9)	7 (77.8)	2 (22.2)

In TSA cases, the most commonly used method for intraoperative skull base repair was tissue glue (*n* = 110/159, 69%): Tisseel (Baxter, Illinois, USA) (*n* = 35/110, 32%), Adherus (Stryker, Michagan, USA) (*n* = 30/110, 27%), Duraseal (Integra Lifesciences, New Jersey, USA) (*n* = 25/110, 23%), Bioglue (Cryolife, Georgia, USA) (*n* = 9/110, 8%), and Evicel (Ethicon, New Jersey, USA) (*n* = 11/110, 10%). Grafts were used in 77 cases (*n* = 77/159, 48%). The type of graft used was tissue (*n* = 32/77, 42%), synthetic (*n* = 31/77, 40%) or both (*n* = 14/77, 18%). When a tissue graft was used, the materials used included fat (*n* = 42/46, 91%), mucosa (*n* = 3/46, 7%), fascia (*n* = 2/46, 4%), bone (*n* = 3/46, 7%), and muscle (*n* = 1/46, 2%). The most common donor site for the tissue graft was the abdomen (*n* = 40/42, 95%). A Spongostan (Ethicon, New Jersey, USA) synthetic graft was used in 40 cases (*n* = 40/45, 89%), Tachosil (Baxter, Illinois, USA) was used in 4 cases (*n* = 4/45, 9%), and Gelfoam (Pfizer, New York, USA) was used once (*n* = 1/45, 2%). Twenty-eight cases (*n* = 28/159, 18%) used dural replacements (a substitute material used specifically to reconstruct the dura, bridging gaps and adding structural integrity) such as Duragen (Integra Lifesciences, New Jersey, USA) (*n* = 21/28, 75%), Duramend (Collagen Matrix, New Jersey, USA) (*n* = 5/28, 18%), and endogenous tissue from the thigh (*n* = 2/28, 7%). In no case was the dura closed directly using sutures or clips. Vascularized flaps were used in 30 cases (*n* = 30/159, 19%), with 22 (*n* = 22/30, 73%) using a nasoseptal flap, 6 (*n* = 6/30, 20%) using a sphenoid mucosa flap, 1 (*n* = 1/30, 3%) using a mucoperichondrial flap, and 1 (*n* = 1/30, 3%) using a middle turbinate flap. Several hemostatic agents (*n* = 82/159, 52%) were used, such as Surgiflo (Ethicon, New Jersey, USA) (*n* = 30/82, 37%), Surgicel (Ethicon, New Jersey, USA) (*n* = 28/82, 34%), Fibrilar (Ethicon, New Jersey, USA) (*n* = 17/82, 21%), Floseal (Baxter, Illinois, USA) (*n* = 14/82, 17%), Lysosypt (B Braun, Meisungen, Germany) (*n* = 1/82, 1%), and Haemopatch (Baxter, Illinois, USA) (*n* = 1/82, 1%).

In terms of EEA cases, the most commonly used method for intraoperative skull base repair was tissue glue (*n* = 22/28, 79%): Tisseel (*n* = 8/22, 36%), Evicel (*n* = 5/22, 23%), Adherus (*n* = 6/22, 27%), and Duraseal (*n* = 3/22, 14%). Grafts were used in 17 cases (*n* = 17/28, 61%). The type of graft used was tissue (*n* = 7/17, 41%), synthetic (*n* = 6/17, 35%), or both (*n* = 4/17, 24%). When a tissue graft was used, the materials used included fat (*n* = 9/11, 82%), fascia (*n* = 6/11, 55%), periosteum (*n* = 1/11, 9%), and bone (*n* = 1/11, 9%). A Spongostan synthetic graft was used in 8 cases (*n* = 8/10, 80%), and Tachosil was used in 2 cases (*n* = 2/10, 20%). Ten cases (*n* = 10/28, 36%) used a dural replacement: Duragen (*n* = 7/10, 70%), Duraform (Natus Medical, California, USA) (*n* = 1/10, 10%), Tutoplast (RTI Surgical, Illinois, USA) Fascia Lata (*n* = 1/10, 10%), and Fascia Lata (*n* = 1/10, 10%). In no case was the dura closed directly using sutures or clips. Vascularized flaps were used commonly (*n* = 21/28, 72%), with 19 (*n* = 19/21, 90%) using a nasoseptal flap, 1 (*n* = 1/21, 5%) using a mucoperichondrial flap, and 1 (*n* = 1/21, 5%) using a sphenoid mucosa flap. Several hemostatic agents (*n* = 21/28, 76%) were used such as Surgicel (*n* = 13/21, 62%), Surgiflo (*n* = 4/21, 19%), Floseal (*n* = 3/21, 14%) and Haemopatch (*n* = 1/21, 5%).

Support to the skull base reconstruction was provided by buttresses and/or nasal packing, which were not directly part of the skull base reconstruction but rather provided external structural stability to the construct. For TSA, a buttress was used in 15 cases (*n* = 15/159, 9%): bone was used 7 times (*n* = 7/15, 47%), Spongostan was used 7 times (*n* = 7/15, 47%), and Medpor (Stryker, Michagan, USA) was used once (*n* = 1/15, 7%). Nasal packs were used in 99 cases (*n* = 99/159, 63%) that used TSA. The types of nasal packs used were Nasopore (Stryker, Michagan, USA) (*n* = 77/99, 78%), Merocel (*n* = 18/99, 18%), and Bismuth Soaked Ribbon Gauze (Martindale Pharma, Buckinghamshire, UK) (*n* = 8/99, 8%). Regarding EEA, a buttress was used in 4 cases (*n* = 4/28, 17%): polyethylene (Medpor) was used twice (*n* = 2/4, 50%), bone was used once (*n* = 1/4, 25%) and Spongostan was used once (*n* = 1/4, 25%). Similarly, nasal packs were used in 26 cases (*n* = 26/28, 92%). The types of nasal packs used were Nasopore (*n* = 20/26, 77%), Merocel (*n* = 5/26, 19%), Bismuth Soaked Ribbon Gauze (*n* = 2/26, 8%), Foley catheter (*n* = 2/26, 8%), and Rapid Rhinos (ArthroCare Corporation, California, USA) (*n* = 2/26, 8%).

### CSF Diversion

A method of CSF diversion was used in 22 cases (*n* = 22/187, 12%): 20 cases (*n* = 20/22, 91%) that used a lumbar drain (TSA: 11/159, 7%; EEA: 9/28, 32%), 1 case that used a ventriculoperitoneal shunt (TSA case), and 1 case that used an external ventricular drain (EEA case). Of the 20 lumbar drains, 5 were continuously clamped postoperatively and removed on day 2 postoperatively, so they in effect did not divert CSF. The 15 remaining lumbar drains remained in situ for a median of 5 days (range, 2–7 days); 4 days (range, 2–6 days) for the TSA group, and 5 days (range, 2–7 days) for the EEA group.

### Postoperative Management

The median length of patient stay was 4 days (range, 1–32 days) for the entire group, 3 days (range, 1–32 days) for the TSA group, and 7.5 days (range, 1–20 days) for the EEA group. Conservative measures to reduce the risk of CSF leak were not specified in 37 cases (*n* = 37/187, 20%). Most patients (TSA: 123/159, 78%; EEA:19/28, 64%) were advised to avoid straining. Medical therapies to prevent or treat postoperative CSF leak were prescribed in 60 patients (*n* = 60/187, 32%), including stool softeners (TSA: *n* = 37/159, 23%; EEA: *n* = 4/28, 14%), prophylactic antibiotics (TSA: *n* = 11/159, 7%; EEA= 8/28, 29%), acetazolamide (TSA: *n* = 0/159, 0%; EEA= 1/28, 4%), and pneumococcal vaccine (TSA: *n* = 0/159, 0%; EEA= 2/28, 7%).

### Postoperative Complications

Overall, 36 patients (*n* = 36/187, 19%) had postoperative complications. The most common complications were diabetes insipidus (TSA: *n* = 6/159, 4%; EEA: *n* = 7/28, 25%), postoperative CSF rhinorrhea (see later discussion) (TSA: *n* = 6/159, 4%; EEA: *n* = 2/28, 7%), and syndrome of inappropriate antidiuretic hormone secretion (TSA: *n* = 4/159, 3%; EEA: *n* = 1/28, 4%). Other complications involving cases that used TSA included meningitis (*n* = 1/159, 1%), sellar abscess (*n* = 1/159, 1%), pneumonia (*n* = 1/159, 1%), mono-ocular blindness (*n* = 1/159, 1%), unspecified hyponatremia (*n* = 1/159), and unspecified hypernatremia (*n* = 1/159, 1%). Among cases that used EEA, other complications included residual disease (*n* = 1/28, 4%), meningitis (*n* = 1/28, 4%), unspecified hyponatremia (*n* = 1/28, 4%) and unspecified hypernatremia (*n* = 1/28, 4%).

### Postoperative CSF Rhinorrhea

Cases of postoperative CSF rhinorrhea (*n* = 8; TSA: 6/159, 3.8%; EEA: 2/28, 7.1%) took a median length of 2 days postoperatively to be reported (range, 1–17 days). Two of these cases were in individuals with BMI >30 (TSA: 1/6, 1/2 EEA). In terms of intraoperative CSF leak, 4 cases had no leak reported (TSA: 4/6, EEA 0/2), there were no cases with grade 1 leak, 2 cases were grade 2 leak (TSA: 2/6, EEA 0/2), and 2 cases were grade 3 leak (TSA: 0/6, EEA 2/2). Two TSA cases used CT scanning of the head (looking for pneumocephalus) as a diagnostic adjunct to β_2_-transferrin. Overall, 6 cases (TSA: 4/6, EEA 2/2) required a return to theater for operative management (CSF diversion, *n* = 1; direct repair, *n* = 1; both, *n* = 4) (Table 4).

**Table 4:** Case Series of Patients with Postoperative Cerebrospinal Fluid Rhinorrhea that Were Confirmed or Required Intervention: Baseline and Tumor Characteristics, Intraoperative Technique and Recognition of Postoperative Cerebrospinal Fluid Rhinorrhea

Case Number	Age (years), Sex	Body Mass Index >30 kg/m^2^	Tumor Type	Tumor Diameter >1 cm	Operative Approach	Dural Defect (cm)	Intraoperative CSF Leak Grade	Intraoperative Repair	Postoperative CSF Rhinorrhea	Return to Theater
1	38, male	Yes	Nonfunctioning pituitary adenoma	Yes	TSA	<1	0	Tisseel, Nasopore	2 days postoperatively (via β_2_-transferrin)	No (conservative management)
2	31, male	No	Dermoid cyst	Yes	EEA	1–3	3	Pedicled NS flap, Spongostan, Tisseel, Nasopore + Merocel	6 days postoperatively (via β_2_-transferrin)	Yes (direct repair)
3	60, female	No	Lung metastasis	Yes	EEA	Not recorded	3	Duragen, fascia lata graft, Nasopore	2 days postoperatively (via β_2_-transferrin)	Yes (lumbar drain)
4	51, male	Yes	Nonfunctioning pituitary adenoma	Yes	TSA	1–3	0	Fat graft, Spongostan, Duraseal, Surgiflo	1 day postoperatively (via β_2_-transferrin and CT head)	Yes (lumbar drain and direct repair)
5	10, female	No	Craniopharyngioma	Yes	TSA	1–3	2	NS flap, Tisseel, Surgicel, Spongostan, Nasopore	9 days postoperatively (via β_2_-transferrin)	Yes (lumbar drain and direct repair and ventriculoperitoneal shunt)
6	30, male	No	Arachnoid cyst	Yes	TSA	1–3	2	Duragen, NS flap, Tisseel, Nasopore	17 days postoperatively (via β_2_-transferrin and CT head)	Yes (lumbar drain and direct repair)
7	76, male	No	Sinonasal carcinoma	Yes	TSA	>3	0	Mucoperichondrial flap, pericranial fascia graft, Tachosil, bone buttress, Sinofoam pack	1 day postoperatively (via clinical assessment alone)	Yes (lumbar drain and direct repair)
8	43, female	No	Nonfunctioning pituitary adenoma	Yes	TSA	<1	0	Surgiflo	2 days postoperatively (via β_2_-transferrin)	No (conservative management)

CSF, cerebrospinal fluid; TSA, transsphenoidal approach; EEA, expanded endonasal endoscopic approach; NS, nasoseptal.

### Qualitative Feedback

Qualitative feedback was collected from 4 pilot leads in phase 1 of the study, informing improvements for phase 2. The content analysis generated 14 codes, refined into 6 themes (Supplementary Table 1). These themes were then categorized into “positives” and “challenges.”

Three principal positives were highlighted. First, the data collection interface was complimented: the Castor software was described as “really simple to use, speeds up data collection and is enjoyable to use”, and the organization of the data proforma (via logic trees) facilitated efficient data entry: “Not overwhelming the user with all the unnecessary questions (and only loading them if needed)” and the “flow is logical.” This collection process was complemented by electronic medical record systems at all pilot centers, allowing pilot leads to establish flexible routines: “15 minutes work a week” with “all electronic notes making the data collection very straight forward.” In addition, supportive materials provided to local teams were applauded for their usefulness (sample audit registration forms, study protocol, practical step-by-step guide, and skull base methods explanatory diagrams). Comments included “excellent diagrams explaining the technical nuance of skull base surgery” and “registration was easy because I had a template to follow.” Pilot leads were generally met with receptiveness from senior colleagues; one pilot lead organized a meeting with senior operating members of the team who “amended her operation notes to specifically mention the things I need to collect data on.” This strategy allowed efficient data collection and consistent data verification.

Local team engagement is crucial to the effective execution of the project. Lapses in this engagement have the potential to present challenges; one pilot lead highlighted “op notes contain limited information, often standardized text” and that it can be “difficult to get a hold of consultants or StR's [specialist trainees] to check with them the data points that need to be checked with them or that weren't clear.” Several approaches were adopted in response to this challenge; one pilot lead met with operating surgeons early on to adapt operative notes to include additional CRANIAL data points (e.g. CSF leak grade and dural defect size), whereas another pilot lead compiled data points needing verification into a table for weekly verifications with operating surgeons. Moreover, the volume and complex nature of the data posed a challenge initially. There was heterogeneity in the definitions and categorization for different skull base repair techniques across centers; to address this, a taxonomy diagram, definitions set, and explanatory illustrations were generated as discussed earlier.^24^ Specific data points were adjusted and clarified based on feedback (e.g. “size of skull base defect” was refined to “max diameter of dural defect,” with categorical answer options [and a “not available” option for instances where this was difficult to ascertain]). Concerns over future compliance with detailed follow-up data were raised; these data points were rationalized and many were made optional to capture primary outcomes without overloading data collectors. The final set of challenges were concerning the future of the project in the context of the coronavirus disease 2019 (COVID-19) pandemic and its impact on endonasal surgery. Guidance for a significant reduction in the amount of endonasal skull base cases was released just after the completion of pilot phase 1 data collection. Resultantly, COVID-19 Crelated data points were added to the data proforma for Phase 2 piloting (reported elsewhere).

## Discussion

### Principal Findings

This pilot study has shown the acceptability and feasibility of the current CRANIAL protocol.^28^ Acceptability is shown through qualitative feedback from local pilot leads, which was largely positive (user-friendly and efficient data collection, felt supported by central CRANIAL team and seniors). Challenges were addressed iteratively (production of supportive materials and adaptations of data proforma), again met positively by pilot leads. Moreover, feasibility is highlighted through the successful registration and execution of the study at 12 tertiary neurosurgical centers, with high-quality data collected on 187 patients.

As expected, most of these endonasal cases were pituitary adenomas (*n* = 142/187, 76%) and the most common approach was TSA (*n* = 159/187, 85%). Although our pilot sample is too small to make conclusions, it is interesting to note the array of repair techniques used. The most common skull base repair techniques used were tissue glues (Tisseel, Adherus, Duraseal, Bioglue, and Evicel) in 132/187 cases (71%), and grafts (most commonly fat graft and Spongostan) in 94/187 cases (50%). These repairs were most frequently supported by nasal packs (Nasopore, Merocel, and Bismuth Soaked Ribbon Gauze) in 125/187 cases (67%). Nasoseptal flaps were used in only 41/187 cases (22%) and lumbar drains were used in 200 /187 cases (11%). Adjuvant conservative and medical prevention of CSF rhinorrhea were equally variable (most commonly laxatives and avoiding straining). The incidence of confirmed postoperative CSF rhinorrhea was 6/159: (3.8%) of TSA cases and 2/28 (7.1%) of EEA cases. In all of these cases, the initial intraoperative skull base repair techniques were heterogeneous. Four of these cases with postoperative CSF rhinorrhea did not have intraoperative CSF leak detected, suggesting occult intraoperative leak. This finding is described in other case series, with some investigators advocating for universal sellar repair or use of routine intrathecal fluorescein to address this.^30^^,^^31^

### Findings in the Context of Literature

In our pilot analysis, the encountered postoperative CSF rhinorrhea rates are in line with the array of rates cited in the literature. For TSA, the occurrence of CSF rhinorrhea is generally between 2% and 5%^7^^,^^8^^,^^20^^,^^21^ but has been recorded as high as 10% via meta-analysis.^32^ Occurrence in EEA is even more diverse (likely reflecting case-specific variations in exact approach), with rates generally ranging from 5% to 20% but as high as 50%.^4^^,^^22^^,^^23^ Risk factors for postoperative CSF rhinorrhea include increased BMI, intraoperative CSF leak (especially if high flow), previous cranial radiotherapy, previous skull base surgery, tumor size, local tumor infiltration, dural defect size, and surgeon experience.^4^^,^^5^^,^^7^^,^^10^, ^11^, ^12^^,^^33^

However, potentially the most important determinant for the development of CSF rhinorrhea is related to skull base repair technique used intraoperatively.^4^^,^^16^ The heterogeneity in skull base repair techniques suggested in our pilot study is echoed in the literature, reflecting the general lack of comparative evidence to guide practice.^14^ Practically, many centers use graded repair protocol–dependent factors such as dural defect size and CSF leak flow volume.^34^ In our series, CSF diversion was used more in the context of tumors >1 cm in diameter, EEA, and high-grade intraoperative CSF leak (Table 3). Similar patterns are noted for the use of vascularized flaps, dural replacement grafts, rigid buttresses, and nasal packing on a basis of such CSF leak risk factors (Table 3).

Several noncomparative studies^17^^,^^18^^,^^23^^,^^34^ have suggested that in the context of large skull base defects (>3 cm) and/or high CSF flow (via the opening of the ventricle or arachnoid cistern), the use of nasoseptal flaps decreases resultant postoperative CSF rhinorrhea. Some investigators advocate for graft-based reconstruction (fat, fascia, and collagen sponge) in this context,^35^^,^^36^ whereas others describe a multifaceted approach combining various techniques (e.g. fat, collagen sponge, rigid buttress, and nasal packs) with or without lumbar drain for high-flow leaks with large dural defects.^16^^,^^34^ The only level 1 evidence supporting practice is a recent randomized controlled trial that found (in the context of dural defects >1 cm^2^ and high-flow intraoperative CSF leak repaired with a nasoseptal flap) that the use of perioperative lumbar drain significantly decreased postoperative CSF rhinorrhea rates (*P* = 0.017; odds ratio, 3.0; 95% confidence interval, 1.2–7.6).^19^ For smaller defects and minor/no CSF leak, fat, fascia, and avascular mucosal grafts are described.^23^^,^^36^ Other repair protocols support the use of collagen sponge and titanium mesh buttress for such cases.^16^ More generally, some surgeons champion dural closure or dural replacements,^37^^,^^38^ with others suggesting that these have little impact in the context of nasoseptal flap use.^39^ Similarly, high-level evidence for postoperative CSF leak repair is equally scarce, with lumbar drains and endonasal direct pedicled flap or graft repair frequently reported.^40^, ^41^, ^42^ There is widespread variability in skull base repair protocols; this is the circumstance in both high and low CSF flow situations, and in both prevention and repair CSF rhinorrhea.^14^

### Limitations

There are several limitations to the study, calling for a tempered assessment of findings. Firstly, because of the pilot nature of this study, results are of small sample size, particularly with respect to EEA. Cases were not necessarily collected consecutively and because of recency of cases, follow-up is limited to the immediate postoperative period (the national project will include up to 6 months of follow-up per case). Data points are purely observational and across the context of multiple centers. Practically, data point verification was sometimes a challenge logistically for junior members of the team, although ways to mitigate this have been presented and will be useful when scaling up this project. One such data point was dural defect, which was not recorded in approximately 30% of cases and in the context of TSA was recorded to include sellar dura (a defect that may not confer the same risk of postoperative CSF leak as dural/arachnoid defects elsewhere).

## Conclusions

Our pilot experience highlights the acceptability, feasibility, and scalability in the CRANIAL project procedures. Early results suggest heterogeneity in methods used for skull base repair. There is a clear precedent for establishing a benchmark of contemporary practice in skull base neurosurgery in the United Kingdom and Ireland via multicenter dissemination of this project.

## CRediT authorship contribution statement

**Danyal Z. Khan:** Conceptualization, Resources, Data curation, Formal analysis, Writing - original draft, Writing - review & editing. **Hani J. Marcus:** Conceptualization, Formal analysis, Writing - original draft, Writing - review & editing, Supervision. **Hugo Layard Horsfall:** Data curation, Formal analysis, Writing - original draft. **Soham Bandyopadhyay:** Resources, Data curation, Formal analysis, Writing - original draft, Writing - review & editing. **Benjamin E. Schroeder:** Formal analysis, Writing - original draft, Writing - review & editing. **Vikesh Patel:** Resources, Data curation, Formal analysis, Writing - original draft, Writing - review & editing. **Alice O’Donnell:** Writing - original draft, Writing - review & editing. **Shahzada Ahmed:** Resources, Writing - review & editing. **Andrew F. Alalade:** Resources, Writing - review & editing. **Ahmad M.S. Ali:** Resources, Data curation, Writing - review & editing. **Callum Allison:** Resources, Data curation, Writing - review & editing. **Sinan Al-Barazi:** Writing - review & editing. **Rafid Al-Mahfoudh:** Resources, Writing - review & editing. **Meriem Amarouche:** Resources, Writing - review & editing. **Anuj Bahl:** Resources, Writing - review & editing. **David Bennett:** Resources, Writing - review & editing. **Raj Bhalla:** Resources, Writing - review & editing. **Pragnesh Bhatt:** Resources, Writing - review & editing. **Alexandros Boukas:** Resources, Data curation, Writing - review & editing. **Ivan Cabrilo:** Resources, Data curation, Writing - review & editing. **Annabel Chadwick:** Resources, Data curation, Writing - review & editing. **Yasir A. Chowdhury:** Conceptualization, Resources, Data curation, Writing - review & editing. **David Choi:** Resources, Writing - review & editing. **Simon A. Cudlip:** Resources, Writing - review & editing. **Neil Donnelly:** Resources, Writing - review & editing. **Neil L. Dorward:** Resources, Writing - review & editing. **Graham Dow:** Resources, Writing - review & editing. **Daniel M. Fountain:** Conceptualization, Resources, Data curation, Writing - review & editing. **Joan Grieve:** Resources, Writing - review & editing. **Anastasios Giamouriadis:** Resources, Writing - review & editing. **Catherine Gilkes:** Resources, Writing - review & editing. **Kanna Gnanalingham:** Resources, Writing - review & editing. **Jane Halliday:** Resources, Writing - review & editing. **Brendan Hanna:** Resources, Writing - review & editing. **Caroline Hayhurst:** Resources, Writing - review & editing. **Jonathan Hempenstall:** Resources, Writing - review & editing. **Duncan Henderson:** Resources, Data curation, Writing - review & editing. **Kismet Hossain-Ibrahim:** Resources, Writing - review & editing. **Theodore Hirst:** Resources, Data curation, Writing - review & editing. **Mark Hughes:** Resources, Writing - review & editing. **Mohsen Javadpour:** Resources, Writing - review & editing. **Alistair Jenkins:** Resources, Writing - review & editing. **Mahmoud Kamel:** Resources, Writing - review & editing. **Richard J. Mannion:** Resources, Writing - review & editing. **Angelos G. Kolias:** Conceptualization, Resources, Data curation, Writing - review & editing. **Mohammad Habibullah Khan:** Resources, Writing - review & editing. **Mohammad Saud Khan:** Resources, Data curation, Writing - review & editing. **Peter Lacy:** Resources, Writing - review & editing. **Shumail Mahmood:** Resources, Data curation, Writing - review & editing. **Eleni Maratos:** Resources, Writing - review & editing. **Andrew Martin:** Resources, Writing - review & editing. **Nijaguna Mathad:** Resources, Writing - review & editing. **Patrick McAleavey:** Resources, Data curation, Writing - review & editing. **Nigel Mendoza:** Resources, Writing - review & editing. **Christopher P. Millward:** Resources, Data curation, Writing - review & editing. **Showkat Mirza:** Resources, Writing - review & editing. **Sam Muquit:** Resources, Writing - review & editing. **Daniel Murray:** Resources, Data curation, Writing - review & editing. **Paresh P. Naik:** Resources, Data curation, Writing - review & editing. **Ramesh Nair:** Resources, Writing - review & editing. **Claire Nicholson:** Resources, Writing - review & editing. **Alex Paluzzi:** Resources, Writing - review & editing. **Omar Pathmanaban:** Resources, Writing - review & editing. **Dimitris Paraskevopoulos:** Resources, Writing - review & editing. **Jonathan Pollock:** Resources, Writing - review & editing. **Nick Phillips:** Resources, Writing - review & editing. **Rory J. Piper:** Resources, Writing - review & editing. **Bhaskar Ram:** Resources, Writing - review & editing. **Iain Robertson:** Resources, Writing - review & editing. **Elena Roman:** Resources, Data curation, Writing - review & editing. **Peter Ross:** Resources, Writing - review & editing. **Thomas Santarius:** Resources, Writing - review & editing. **Parag Sayal:** Resources, Writing - review & editing. **Jonathan Shapey:** Resources, Writing - review & editing. **Rishi Sharma:** Resources, Writing - review & editing. **Simon Shaw:** Resources, Writing - review & editing. **Alireza Shoakazemi:** Resources, Writing - review & editing. **Syed Shumon:** Resources, Data curation, Writing - review & editing. **Saurabh Sinha:** Resources, Writing - review & editing. **Georgios Solomou:** Resources, Writing - review & editing. **Wai Cheong Soon:** Resources, Data curation, Writing - review & editing. **Simon Stapleton:** Resources, Writing - review & editing. **Patrick Statham:** Resources, Writing - review & editing. **Benjamin Stew:** Resources, Writing - review & editing. **Nick Thomas:** Resources, Writing - review & editing. **Georgios Tsermoulas:** Resources, Writing - review & editing. **James R. Tysome:** Resources, Writing - review & editing. **Adithya Varma:** Resources, Data curation, Writing - review & editing. **Philip Weir:** Resources, Writing - review & editing. **Adam Williams:** Resources, Writing - review & editing. **Mohamed Youssef:** Resources, Writing - review & editing.

## Figures and Tables

**Supplementary Table 1:** Summary of Qualitative Feedback from the Pilot Study Leads–themes and Component Codes

Themes	Codes	No. of pilot leads describing this code	No. of data points within code
Positives			
User-friendly data collection process	Easy Castor software interface	4	5
	Logical data form	1	4
	Efficient data entry	4	6
	Routine establishment	2	3
The utility of supportive materials	The utility of supportive materials	3	4
Receptive senior engagement	Positive experience with seniors	4	5
Challenges			
High volume, complex data points	Difficult taxonomy	4	6
	Unclear/complex data points	3	6
	Follow up data - compliance	1	1
Need for teamwork and senior buy-in	Data verification challenges	2	2
	Amount of data points	1	1
	Difficult offsite data collection	1	1
Pandemic related concerns	COVID-19 impact on case availability	1	2
	COVID-19 data collection	1	1
